# Feasibility pilot of Cooking Together: Preliminary results

**DOI:** 10.1002/alz70858_105684

**Published:** 2025-12-26

**Authors:** Kristina Devlin, Sheng Han Liu, Kate Dupuis, Laura E. Middleton, Heather Keller

**Affiliations:** ^1^ University of Waterloo, Waterloo, ON, Canada; ^2^ Sheridan College, Oakville, ON, Canada; ^3^ Schlegel‐UW Research Institute for Aging, Waterloo, ON, Canada

## Abstract

**Background:**

Community‐based programs, such as intergenerational programs, have been identified as a viable option to reduce dementia stigma and foster connections between generations. Cooking and eating with others is a social opportunity that bring people together. To date, few programs have been developed using food activities to connect community‐dwelling persons living with dementia (PWD) and young adults (18‐30 years old). Cooking Together is an innovative multi‐week intergenerational cooking and nutrition program that was collaboratively developed and evaluated. The aim of this ongoing pilot study is to test a revised program model in three separate offerings (up to 10 participants/offering) to determine feasibility, usability of procedures and outcomes, and program utility. We report preliminary feasibility based on two offerings.

**Method:**

Weekly 2‐hour sessions are chef‐facilitated, using a brain‐health focused menu. Feasibility was evaluated through recruitment (8‐10 eligible participants per offering in 2 months), attendance (70% sessions attended after enrolment), and completion of pre‐ and post‐program evaluation measures (85% participants). Young adults were recruited through a contact list of individuals who expressed interest in a previous offering but did not participate, campus advertisements, and word of mouth. PWD were recruited through a senior day program, repository of PWD interested in research studies, and word of mouth.

**Result:**

Ten young adults (age (median (range)): 19 (18‐30), 90% women, 50% white) and 9 PWD (age (median (range)): 73 (36‐80), 56% women, 89% white) were recruited over two offerings within a two‐month time period. Long lead times to reserve kitchen space and scheduling meant that the offering start dates had limited flexibility, resulting in ongoing recruitment until the second week after the offering started. Fourteen participants (74%; 7 young adults and 7 PWD) completed both pre‐ and post‐program measures. Average attendance at cooking sessions was 87%, with 40% attending the full program. Common reasons for non‐attendance were prior commitments or illness.

**Conclusion:**

Preliminary findings from two offerings indicate that Cooking Together is feasible and acceptable. Recruitment for the third offering is underway, with data collection expected to be completed by April 2025. Findings from this study will inform future efficacy testing.

